# Managing Potential Laboratory Exposure to Ebola Virus by Using a Patient Biocontainment Care Unit^1^

**DOI:** 10.3201/eid1406.071489

**Published:** 2008-06

**Authors:** Mark G. Kortepeter, James W. Martin, Janice M. Rusnak, Theodore J. Cieslak, Kelly L. Warfield, Edwin L. Anderson, Manmohan V. Ranadive

**Affiliations:** * US Army Medical Research Institute of Infectious Diseases, Fort Detrick, Maryland, USA; † San Antonio Military Pediatric Center, San Antonio, Texas, USA

**Keywords:** Ebola virus, Marburg virus, viral hemorrhagic fever, filovirus, biocontainment, quarantine, isolation, biosafety, laboratory exposure, synopsis

## Abstract

In 2004, a scientist from the US Army Medical Research Institute of Infectious Diseases (USAMRIID) was potentially exposed to a mouse-adapted variant of the Zaire species of Ebola virus. The circumstances surrounding the case are presented, in addition to an update on historical admissions to the medical containment suite at USAMRIID. Research facilities contemplating work with pathogens requiring Biosafety Level 4 laboratory precautions should be mindful of the occupational health issues highlighted in this article.

## Abstract 2

One-sentence summary for table of contents: Recommendations are needed for management of potential laboratory exposure to a Biosafety Level 4 pathogen.

Recent interest and increased investment in biodefense research have resulted in construction of new research laboratories with Biosafety Level 4 (BSL-4) capability (*1*). In addition to ensuring biosafety, due consideration should be given to managing medical, public health, and public relations issues related to occupational exposures to highly hazardous infectious pathogens.

We present a potential exposure to Ebola virus that occurred in a BSL-4 laboratory at the US Army Medical Research Institute of Infectious Diseases (USAMRIID). Background and prior use of the medical containment suite (MCS) are reviewed briefly, followed by discussion of pertinent issues related to the event and recommendations for response.

## Case Report

In 2004, a virologist at USAMRIID was working in a BSL-4 laboratory with mice that had been infected 2 days before with a mouse-adapted variant of the Zaire species of Ebola virus (ZEBOV) (*2*). The virulence and infectious dose of this variant of ZEBOV are unknown in humans; wild-type virus has a case-fatality rate of up to 90% (*3*).

The person had been following standard procedure, holding the mice while injecting them intraperitoneally with an immune globulin preparation. While the person was injecting the fifth mouse with a hypodermic syringe that had been used on previous mice, the animal kicked the syringe, causing the needle to pierce the person’s left-hand gloves, resulting in a small laceration. The virologist immediately squeezed the site to force the extravasation of blood. After decontamination of the blue suit in the chemical shower, the injured site was irrigated with 1 liter of sterile water and then scrubbed with povidone-iodine for 10 minutes.

In terms of exposure risk, the needle was presumed to be contaminated with virus-laden blood, although it was suspected that low levels of virus were present on the needle. The animals had not yet manifested signs of infection, and much contamination may have been removed mechanically when the needle pierced the gloves. The local decontamination of the site also reduced potential for infection.

USAMRIID medical, scientific, and executive staff concluded that the person with potential exposure warranted quarantine in the MCS. Contact plus airborne precautions (gown, gloves, N95 mask, eye protection) were used, with a plan to upgrade to BSL-4 precautions for signs or symptoms of illness. These extra precautions were instituted while the patient was asymptomatic for several reasons: 1) the timing of initial clinical manifestations with regard to potential for shedding virus were not known for this specific isolate in human infection; 2) there was interest in ensuring all infection control procedures were being followed appropriately in advance of clinical illness; and 3) there was interest in reducing any potential confounders, such as a caregiver transmitting a febrile respiratory infection to the patient, which might lead to unnecessary procedures or additional isolation. The person was monitored for routine vital signs; daily laboratory studies (coagulation studies, blood counts, chemistries, viral isolation, D-dimer) and regular physician assessments were performed.

Over the next several days, discussions were held with several internationally recognized filovirus experts regarding potential treatments or postexposure prophylaxis options. Local and state public health officials were also notified. The consensus opinion was that there was no safe, readily available source of immune plasma and little evidence existed to support its use. Emergency investigational new drug (IND) protocols were established for treatment with recombinant nematode protein (rNAPc2) and antisense oligomers, with the intention to consider implementation only if the patient demonstrated evidence of infection.

Ultimately, none of the 5 mice had confirmed viremia at the time of the incident. The patient did not become ill or seroconvert and was discharged after 21 days. The story received national and local media attention (*4*,*5*).

## The MCS (“Slammer”)

In planning for USAMRIID (established in 1969), the decision was made to include a maximum containment (now termed BSL-4) capability to care for 2 personnel who may have been exposed to a biologic agent. This MCS would also be available for managing occupational exposures at USAMRIID or other government agencies to diseases requiring containment (R. McKinney, pers. comm.).

The 1,288-square-foot MCS includes 2 patient rooms and a treatment room and is equipped for intensive care monitoring, ventilator use, and teleradiology capability. It has an independent ventilation system and a chemical shower for decontaminating caregivers’ encapsulating suits (identical to those worn in the BSL-4 laboratories), and it is isolated from adjacent areas by doors fitted with airtight gaskets. This latter feature earned the facility the moniker “the slammer,” which was popularized in the book The Hot Zone (*6*).

The MCS is staffed by USAMRIID personnel, with augmentation and specialized care provided by nearby medical center staff, who serve on a medical augmentation team (*7*). Passage of consumables and supplies in and out of the suite occurs through 3 conduits: a double-door autoclave, an ultraviolet light passbox, and a disinfectant dunk tank, which enables decontamination and transport of specimens to external areas within the facility for laboratory analysis. The MCS is maintained under negative pressure. Air undergoes HEPA filtration upon entry to and exit from the facility. The septic system links into USAMRIID’s laboratory sewer system, which undergoes steam sterilization.

## Prior MCS Admissions

Twenty-one patients have been considered candidates for admission to the MCS (Table 1) (*7*,*8*). Eighteen were USAMRIID investigators and 3 were from elsewhere. Four patients (3, 6, 7, and 18) would likely not be admitted today: 2 involved dengue virus (now a BSL-2 pathogen) and 1 each involved Japanese encephalitis B virus and Rift Valley fever virus (BSL-3 pathogens with licensed and investigational vaccines, respectively) (*1*).

**Table 1:** Admissions into the medical containment suite at the US Army Medical Research Institute of Infectious Diseases, 1972–2004*

Patient no.	Date of admission	Days in isolation	Virus†	Reason for admission	Therapy‡	Comments§
1	1972 Oct	18	Machupo	Cut finger	IP	
2	1975 Oct	42	Machupo	Cut finger	IP, IG	
3	1976 Oct	21	JEB	Fingerstick		
4	1977 Sep	14	Machupo	Vial leak		
5	1977 Sep	14	Machupo	Vial leak		
6	1978 May	11	Dengue	Not specified		Modified CC
7	1978 May	8	Dengue	Not specified		Modified CC
8	1978 Jun	17	Lassa	Dropped vial	LIG	
9	1978 Jun	17	Lassa	Dropped vial	LIG	
10	1978 Jul	8	Lassa	Field exposure		
11	1978 Nov	14	Lassa	Suit seam failed		
12	1979 May	20	Lassa	Fingerstick	IP	
13¶	1979 Jul	21	Lassa	Fingerstick	IP	
14	1979 Nov	20	Lassa	Fingerstick	IP, Rib	
15	1981 May	14	Ebola/Lassa	Field exposure		Modified CC
16	1982 Oct	14	Junin	Defective suit seal		Conventional
17	1982 Dec	21	Junin	Fingerstick	IP	
18	1983 Jan	3	Rift Valley fever	Waste exposure		
19	1983 Apr	14	Junin	Defective suit seal		Conventional
20	1985 May	4	Junin	Fingerstick		
21	2004 Feb	21	Ebola	Fingerstick		

*Modified from Cieslak et al. (*8*) with permission. †JEB, Japanese encephalitis virus B; Ebola/Lassa, potential exposure to these viruses. ‡IP, immune plasma from previously infected survivors; IG, immune globulin; LIG, Lassa immune globulin; Rib, ribavirin. §CC, containment care; modified CC, provided by converting a separate physical facility into a Biosafety Level 4–like suite; conventional, Biosafety Level 3 isolation was permitted for 2 lower risk exposures. ¶Not noted in previous reports (*7*,*8*).

Three patients (6, 7, and 15) were managed in an improvised manner. The MCS was unavailable for 1 patient because the unit was undergoing maintenance. Thus, the observation was conducted in another set of rooms without high-level containment features. Two other patients (16 and 19) were deemed low risk; consequently, isolation was permitted under more conventional conditions.

Of the remaining 14 admissions after potential exposure to BSL-4 viruses, 8 involved percutaneous injury and 6 involved potential aerosol exposure. Eight persons (5 evaluated for exposure to Lassa virus, 2 for Machupo virus, and 1 for Junin virus) received immune plasma (prepared from recovered patients in virus-endemic areas); 1 patient potentially exposed to Lassa virus also received intravenous ribavirin. No patient developed disease or seroconverted. From 1985 through 2003, no potential exposure in a USAMRIID BSL-4 laboratory was deemed a high enough risk to require quarantine.

## Discussion

Laboratories remain a potential venue for exposures to BSL-4 viruses (*1*). Filoviruses, in particular, have been associated with laboratory-acquired infection, being first identified after exposure to African green monkeys in Marburg, Germany, in 1967 (*9*). Since then, laboratory-acquired Ebola virus infections have occurred in England (*10*) and Côte d’Ivoire (*11*). The death of a Russian researcher in 2004 (*12*) from laboratory-acquired Ebola virus infection and USAMRIID’s recent experience demonstrate the seriousness of this issue.

Although USAMRIID periodically manages potential laboratory exposures (*13*–*16*), potential exposures in BSL-4 laboratories are rare. Nonetheless, as more facilities conduct research on viruses requiring BSL-4 containment, cases such as the one presented herein may become more commonplace.

The decision to place someone in quarantine is a difficult one. When this potential exposure occurred, several alternatives were considered: sending the patient home with periodic home or clinic assessments, admission to a medical center, and admission to the MCS. Some less serious potential exposures had been managed with twice-a-day vital sign assessments and exclusion from the laboratory. The current situation appeared to present a much higher risk to the person than past potential exposures: the patient had a break in the skin caused by a potentially contaminated needle, a route of infection known to transmit efficiently and associated with enhanced risk for death (*17*). Therefore, the most reasonable approach was determined to be quarantine in the MCS, thus enabling closer monitoring than could be provided at home and ensuring the safety of caregivers and family members.

Quarantine in a hospital was considered. This option presents certain safety challenges in an unprepared facility, including safe handling, transport, and analysis of laboratory specimens within the hospital; safe disposal of waste; potential reluctance of hospital staff (unfamiliar with viral hemorrhagic fevers) to care for such an infected person; and lack of a specific area within the hospital configured for handling of this type of patient.

There are advantages and disadvantages to using the MCS as a stand-alone medical facility. It enables close monitoring separate from other patients (thus eliminating risk of nosocomial spread and cross-contamination); its personnel are already trained in managing a patient in containment; public access is limited; a proven system is in place for waste disposal; and an on-site containment laboratory (with the ability to culture virus or perform sophisticated diagnostic testing under containment conditions) reduces risk for infection of clinical laboratory personnel and contamination of laboratory equipment. Disadvantages of a stand-alone facility include lack of ready access to consultative physicians; critical care nursing; radiologic and other imaging studies; blood products, medications, and resuscitative procedures; and other services available at a large medical center. Activities undertaken to compensate for these deficiencies include staffing the MCS with intensive care and infectious disease physicians and having other consultants available, as needed. Moreover, ventilator and dialysis machines and blood product and laboratory support can be kept on stand-by status within or near the facility. However, it is easier to have an isolation unit located with or within a major medical center, as has been conducted elsewhere (*18*).

One might question whether a facility such as the MCS is the most appropriate place to isolate an infected patient. The US Centers for Disease Control and Prevention (CDC) advises that patients with viral hemorrhagic fevers can be managed safely in a conventional hospital with, at the most, airborne and contact precautions (*19*–*21*). Those patients with potential exposures (close or high-risk contacts of infected persons) who are not ill should be placed under surveillance with twice-a-day temperature checks, and a period of observation is appropriate (*19*). However, setting(s) for this observation period were not specified. Home observation works well for potential exposures deemed low risk; however, waiting for a patient at higher risk for highly hazardous or contagious diseases to manifest fever at home is not ideal.

The MCS was constructed with the premise that certain rare situations might call for extraordinary precautions to isolate victims of severe contagious diseases. These precautions reduce risk for a virus such as Ebola being introduced into the community by minimizing risk for nosocomial spread and optimizing known effective infection control practices. Although these precautions are useful for filoviruses, they may be more useful for other viral hemorrhagic fevers that are transmitted more readily by the aerosol route or are potentially adaptable to local animal reservoirs. The facility may provide some reassurance to the community (and thus serve to lessen public anxiety related to a filovirus exposure) and to laboratory researchers that there is a place for their care if they become infected. A patient with a filovirus infection in an unprepared medical facility would be handled as safely as possible, using CDC guidelines (if the disease were recognized). It is acknowledged that BSL-4–like infection control precautions may not be necessary for observation or illness. However, most clinical experience managing filovirus infections is from sub-Saharan Africa, where increased temperature and humidity may reduce stability of viruses in aerosol (*22*). Contrast that environment with a US hospital where air is cool, dry, and recycled within the facility, a setting potentially more conducive to airborne virus spread.

A laboratory-acquired case of Sabia virus provides an example of how a patient infected with a BSL-4 agent might be managed safely in a community hospital but also provides cause for caution and heightened vigilance, especially among facilities that might receive patients referred from containment laboratories (*23*). The Sabia virus–infected scientist did not report the initial potential exposure and only came to the attention of healthcare providers after 5 days of illness. He was evaluated initially at a tropical medicine clinic and was subsequently referred to an emergency department. Although the department was notified that the arriving patient might have been infected with an arenavirus, there was a 12-hour delay before heightened infection control measures (specific for managing a viral hemorrhagic fever patient) were instituted. A total of 142 persons were identified as potential case-contacts, including 61 workers in the hospital clinical laboratory. Although no secondary cases occurred, potential risk and anxiety of contacts, as well as costs of an investigation by 3 agencies (CDC, the Connecticut Department of Public Health, and Yale University) and a 6-week period of surveillance, argue in favor of 1) an aggressive program of reporting and evaluating any mishap or potential exposure occurring in a containment laboratory; 2) use of facilities familiar with and prepared in advance, when possible, for managing a similar patient; and 3) a preestablished method for surveillance and site for potential quarantine of high-risk exposures.

USAMRIID is not unique in foreseeing the need for a special isolation unit. Emory University and the University of Nebraska maintain special isolation wards for patients with potentially contagious, highly hazardous diseases, and recommendations on design and planning for biocontainment patient care units have recently been published (*18*).

In our recent case, the patient voluntarily entered the MCS. Had the patient refused to be quarantined, decision and authority on forcible quarantine would have rested with local or state health departments. Although there is some variability in local and state regulations, authority to enforce isolation and quarantine derives from the states’ power to “safeguard the health, safety, and welfare of its citizens” (*24*).

There are no approved treatments or postexposure prophylaxis regimens for filovirus infections. Use of passive immunotherapy was considered; however, no studies support a definite benefit, and no readily available safe source of such products exists. On the basis of limited data indicating improved survival in rhesus macaques challenged with ZEBOV and treated with rNAPc2, an emergency IND protocol was obtained for using rNAPc2 (*25*). Another emergency IND protocol was obtained for use of antisense oligonucleotides on the basis of demonstrated safety with these compounds for other indications (*26*). Use of either protocol was without proven safety or efficacy in Ebola virus–infected humans. Both products were available for therapeutic use had the patient developed infection with clinical manifestations that warranted aggressive treatment.

Subsequent studies have demonstrated promise for treatment of ZEBOV infections with antisense oligonucleotides and small interfering RNAs (*27*,*28*). An effective vaccine would reduce inherent hazards in working with these viruses. There have been some recent developments with virus-vectored vaccines with and without naked DNA vaccine priming (*29*–*31*). A phase I human study of such a vaccine is ongoing (*32*).

## Management Considerations

Given increasing interest in construction of additional laboratories for study of BSL-4 agents, potential exists for clinicians to manage an occupational exposure to these viruses. Our experience led us to formulate a stepwise approach that might help others plan for and manage similar incidents.

### Step 1: Prepare

Occupational health clinics associated with containment laboratories should develop methods of assessing need for isolation and laboratory decontamination, exit, and notification procedures. Maintaining a close relationship with the biosafety office, thereby knowing the agents in use, will make planning appropriate treatments in advance easier.

It should be determined in advance where an asymptomatic patient might be observed and where to isolate and treat an infected patient. Separate locations may be required, but moving an ill patient may be challenging. Thus, memoranda of understanding must be established in advance that articulate each facility’s role. One should also have 24-hour recall rosters of key personnel that are used occasionally.

### Step 2: Assess the Patient

A primary physician should be designated to develop the treatment/isolation plan in consultation with other experts. New diseases or medications need to be queried at the time of exposure evaluation if employees did not previously notify occupational health officials. This information must be gathered in a nonpunitive environment so that reporting of potential exposures is not discouraged. Details of the exposure incident should be obtained from the patient, the patient’s supervisor, department chief, and laboratory co-workers.

Risks for exposure and disease should be estimated with available information as reported (*13*,*14*). Care for family members, including children, the elderly, or pets, may need to be addressed, in addition to issues such as powers of attorney, advanced directives, last wills and testaments, and similar legal matters.

### Step 3: Gather Appropriate Consultants and Team

Designating another person to coordinate other activities surrounding a high-profile exposure (arranging conferences with external experts, handling media inquiries, issuing press releases, and interacting with external agencies) frees the primary physician to care for the patient. For any clinically important exposures, especially in the absence of licensed therapeutics, it is appropriate to seek advice of consultants (Table 2). These persons may vary, depending on the organization, the pathogen in question, and individual expertise.

**Table 2:** Consultants to consider for establishing a team to manage a potential laboratory exposure

Consultant no.	Title and description
1	Designated primary physician
2	Scientific expert: This person knows the latest medical/scientific literature on the organism.
3	Director of safety: This person will assess the mechanism of injury and how to avoid a repeated occurrence.
4	Research institute or laboratory director’s representative: This person may serve as the liaison to external political, media, or scientific agencies and will need to be aware of the progress of the patient or any investigation to convey accurate information externally and internally.
5	Patient’s supervisor or department chief: This person will need to reassess the specific laboratory methods used (in conjunction with safety) and modify procedures as needed.
6	Representative from regulatory affairs: This person may serve as a liaison to regulatory agencies such as the Food and Drug Administration, especially if establishing an emergency investigational new drug protocol is contemplated.
7	Public affairs representative: This person needs accurate information to hold press briefings or to generate press releases.
8	Occupational health representative: This person should work in conjunction with safety experts to analyze the mechanism of exposure and ways to prevent a recurrence.
9	Scribe: This person will keep track of the key contacts and decisions, as well as the different courses of action considered.
10	Patient: In many cases, the patient may be the most well-informed person on the specific pathogen. His or her level of expertise and interest will determine whether to include the patient in group discussions. If the patient is already in isolation, a family representative may be considered to participate in group discussions with the patient’s approval. Health Insurance Portability and Accountability Act* privacy regulations still apply.

*Public Law 104-191 Health Insurance Portability and Accountability Act of 1996, August 21, 1996 [cited 2007 Aug 27]. Available from www.hhs.gov/ocr/hipaa

Local and state public health agencies will need to be part of discussions if there is potential public health impact; these organizations will likely be fielding queries from the public and the press simultaneously. Local hospitals should be informed if there is potential for transferring the patient to those facilities. The Food and Drug Administration should be informed if establishment of an emergency use IND is contemplated. Any laboratory that might test clinical samples should also be informed in advance of specimens arriving.

### Step 4: Determine the Appropriate Level of Infection Control Measures

Although specialized containment care procedures and facilities may play a limited role in certain extraordinary cases, such as those discussed here, CDC has published guidance for management of viral hemorrhagic fevers in more conventional settings (*19*–*21*). Standard, contact, and droplet precautions and a private room are recommended in initial outpatient or inpatient assessments in early stages of illness, and a face mask should be placed on patients with respiratory symptoms. A room capable of airborne isolation should be considered early to prevent later need for transfer. Precautions should be upgraded to airborne isolation if a prominent cough, vomiting, diarrhea, or hemorrhage develop in a patient, or if the patient undergoes procedures that may stimulate coughing or generation of aerosols.

### Step 5: Provide Additional Communications

Because filovirus exposure has a particular cachet and media interest may be intense, it is preferable to inform the media proactively. Public affairs personnel will need to develop press releases and arrange interviews in conjunction with a medical or scientific expert. Lessons can be learned from the negative publicity received after the tularemia exposures at Boston University (*33*,*34*) and the death of the Russian researcher from infection with Ebola virus (*12*) after a delay in disseminating that information.

Regular communication with the laboratory’s workforce should be maintained. Medical care personnel also need regular updates on modifications of procedures and ongoing reemphasis of infection control practices.

### Step 6: Conduct Appropriate Isolation Logistics

A patient in quarantine results in logistical challenges (providing food and equipment and decontaminating personal, medical, and food waste) even before illness develops. CDC provides recommendations for specimen handling of viral hemorrhagic fever patients (*19*–*21*) that include 1) minimizing laboratory procedures, 2) alerting the laboratory of the nature of the specimens, 3) transporting specimens in decontaminated leak-proof plastic containers, 4) processing laboratory specimens in a class II biologic safety cabinet with BSL-3 practices, and 5) performing virus isolation or culture in a BSL-4 laboratory. If possible, CDC recommends pretreatment of serum specimens with heat (56°C) combined with polyethylene glycol *p*-tert-octylphenyl ether (Triton X-100) at a concentration of 10 μL/mL of serum to reduce viral titer; however, 100% inactivation may not occur (*21*). Automated analyzers should be cleaned and disinfected according to manufacture recommendations or with sodium hypochlorite at a concentration of 500 ppm (1:100 dilution) (*20*,*21*).

One should also limit the number of staff performing 24-hour monitoring and establish restricted room access and an entry-tracking log. If the patient becomes ill, some staff who entered the room may require illness surveillance, especially if there were any breaches in infection control practices. A visitation policy may need to be addressed. Because spending weeks in quarantine can be particularly stressful for the patient, it is useful to consider ways to keep the patient occupied, such as Internet connectivity, a television/video player, and a telephone.

### Step 7: Decide on Treatment

Decisions on treatment/prophylaxis are difficult for viruses requiring BSL-4 precautions that lack any licensed therapy or prophylaxis. Therefore, having access to subject matter experts (as discussed in step 3) is essential. Collectively, difficult treatment decisions may be required that balance risk from investigational therapies against presumed risk for disease.

### Step 8: Keep a Journal

Designation of a scribe early on should be considered to track major events, decision points, and options that were considered. Records of dates and times of important contacts should be included. Meeting minutes should be generated. Maintaining accurate logs may be useful to defend difficult decisions later and may help drive an after-action review.

### Step 9: Learn from the Experience

It is useful to conduct a formal incident review that assesses how the event was managed. Results from any safety or epidemiologic investigations should be included. With appropriate review of procedures and training, additional potential exposures may be prevented.

## Conclusions

There are few institutions in the United States currently capable of working with viruses that require BSL-4 containment, although the list is expected to expand in the near future. This article highlights medical issues and provides management considerations on the basis of USAMRIID’s experience related to a recent potential exposure to a filovirus. The expectation is that as other facilities contemplate conducting research with BSL-4 pathogens, this report may enable them to improve their preparation for potential exposures in the future.
